# Targeting vertebrate intron-encoded box C/D 2′-O-methylation guide RNAs into the Cajal body

**DOI:** 10.1093/nar/gku287

**Published:** 2014-04-20

**Authors:** Aline Marnef, Patrica Richard, Natalia Pinzón, Tamás Kiss

**Affiliations:** 1 Laboratoire de Biologie Moléculaire Eucaryote du CNRS, UMR5099, IFR109 CNRS, Université Paul Sabatier, 118 route de Narbonne, 31062 Toulouse Cedex 9, France; 2 Biological Research Centre, Hungarian Academy of Sciences, Szeged, Hungary

## Abstract

Post-transcriptional pseudouridylation and 2′-O-methylation of splicesomal small nuclear ribonucleic acids (snRNAs) is mediated by box H/ACA and box C/D small Cajal body (CB)-specific ribonucleoproteins (scaRNPs), respectively. The WD-repeat protein 79 (WDR79) has been proposed to interact with both classes of modification scaRNPs and target them into the CB. The box H/ACA scaRNAs carry the common CAB box motif (consensus, ugAG) that is required for both WDR79 binding and CB-specific accumulation. Thus far, no *cis*-acting CB-localization element has been reported for vertebrate box C/D scaRNAs. In this study, systematic mutational analysis of the human U90 and another newly identified box C/D scaRNA, mgU2-47, demonstrated that the CB-specific accumulation of vertebrate intron-encoded box C/D scaRNAs relies on GU- or UG-dominated dinucleotide repeat sequences which are predicted to form the terminal stem-loop of the RNA apical hairpin. While the loop nucleotides are unimportant, the adjacent terminal helix that is composed mostly of consecutive G.U and U.G wobble base-pairs is essential for CB-specific localization of box C/D scaRNAs. Co-immunoprecipitation experiments confirmed that the newly identified CB localization element, called the G.U/U.G wobble stem, is crucial for *in vivo* association of box C/D scaRNPs with WDR79.

## INTRODUCTION

Eukaryotic ribosomal ribonucleic acids (rRNAs) and spliceosomal small nuclear RNAs (snRNAs) undergo extensive post-transcriptional 2′-O-ribose methylation and pseudouridylation during maturation (1,2). The unique physicochemical properties of 2′-O-methylated ribonucleotides and pseudouridines contribute to the correct assembly and function of eukaryotic ribosomes and spliceosomes (3–10).

Site-specific 2′-O-methylation and pseudouridylation of rRNAs and snRNAs are mediated by box C/D and H/ACA modification guide ribonucleoproteins (RNPs), respectively (reviewed in 11–15). Each modification guide RNP is composed of a specific box C/D or H/ACA guide RNA and at least four common C/D (fibrillarin, 15.5 kDa, Nop56 and Nop58) or H/ACA (dyskerin, Nop10, Nhp2 and Gar1) core proteins. The box H/ACA pseudouridylation guide RNAs fold into the evolutionarly conserved hairpin-hinge-hairpin-tail structure, in which the single-stranded hinge and tail regions carry the conserved H (AgAnnA) and ACA boxes (16–18). Apart from the common kink-turn motif formed by the conserved C (RUGAUGA) and D (CUGA) boxes and the flanking 5′- and 3′-terminal sequences, the 2′-O-methylation guide RNAs show no overall structural conservation (19–21). Both classes of guide RNAs carry short target recognition sequences which transiently base-pair to the complementary rRNA and snRNA sequences to position the selected substrate ribonucleotide at the catalytic centre of the guide RNA-associated methyltransferase (fibrillarin) or pseudouridine synthase (dyskerin) (reviewed in 22–26).

Co-transcriptional pseudouridylation and 2′-O-methylation of eukaryotic rRNAs occurs within the nucleolus where nascent pre-rRNAs are synthesized and ribosomal modification guide RNPs, called the small nucleolar RNPs (snoRNPs), accumulate. In contrast, guide RNPs directing pseudouridylation and 2′-O-methylation of Pol II-synthesized spliceosomal snRNAs concentrate in the nucleoplasmic Cajal bodies (CBs) and they are termed small CB-specific RNPs (scaRNPs) (27–29). CBs are evolutionarily conserved, dynamic, multifunctional subnuclear structures which participate in the assembly of small RNPs involved in pre-messenger RNA (mRNA) splicing, ribosome biogenesis, histone mRNA processing and telomere synthesis (30–35). CBs are most prominent in transcriptionally active cells, including muscle, neuronal, embryonic and cancer cells and they likely provide accelerating platforms for RNA modification and RNP assembly reactions which probably can also occur in the soluble fraction of the nucleoplasm (36,37). ScaRNP-mediated modification of pre-snRNAs occurs in the CBs after cytoplasmic assembly and nuclear re-importation of the nascent core snRNPs (38,39). Modification of snRNAs promotes the last step of snRNP biogenesis, the assembly of snRNPs with specific RNP proteins (3,40).

The nucleolar localization of box C/D and H/ACA snoRNPs is supported by the conserved box C/D and H/ACA RNA motif and the associated box C/D and H/ACA core proteins (20,41–44). The molecular mechanism responsible for targeting scaRNPs into the CB is less understood. Recently, the WD-repeat protein 79 (WDR79) has been identified as a scaRNA-associated protein that is required for CB-specific accumulation of both box C/D and H/ACA scaRNPs (45–47). Box H/ACA scaRNAs carry an universally conserved CB localization signal sequence, the CAB box (consensus: ugAG), located in the terminal loop of the 5′ and/or 3′ hairpins of the RNAs (45,48,49). Although indispensable for *in vivo* WDR79 binding, the CAB box alone is unable to tether WDR79 to H/ACA RNAs under *in vitro* conditions (45). This suggests that WDR79 is recruited to H/ACA scaRNPs through forming interactions with the RNA CAB box and the H/ACA core protein(s).

*Drosophila* box C/D scaRNAs carry another CB-targeting sequence that seems to be a 3′-terminally extended version of the H/ACA CAB box (consensus: cgaGUUAnUg) and that alone can specifically interact with WDR79 (45). In mammalian cells, box C/D spliceosomal 2′-O-methylaton guide RNAs are frequently expressed in the form of box C/D-H/ACA double domain RNAs and they utilize the CAB boxes of the H/ACA domains for CB-specific localization (27,48,50). Other mammalian box C/D scaRNAs lacking an H/ACA domain also associate with WDR79, but they possess neither box H/ACA- nor *Drosophila* box C/D-type CB localization sequences (45). In this study, we demonstrate that vertebrate intron-encoded box C/D scaRNAs share similar (GU)-rich dinucleotide repeat sequences which form the terminal stem-loops of the long apical hairpins of the RNAs. Systematic mutational analysis of a newly identified human intron-encoded box C/D scaRNA, mgU2-47 and the previously characterized U90 scaRNA confirmed the importance of the terminal RNA helix that is composed mostly of G.U and U.G wobble pairs both for CB-localization and WDR79-binding.

## MATERIALS AND METHODS

### General procedures

Standard laboratory procedures were used for manipulation of DNA, RNA, oligodeoxynucleotides and proteins (51). Both standard and modified oligodeoxynucleotides containing aminoallyl-T residues were synthesized by Eurofins MWG. HeLa cells were grown in Dulbecco's modified Eagle medium supplemented with 10% fetal calf serum (Invitrogen). Transient transfection of HeLa cells was performed by using jetPRIME (Ozyme) or Lipofectamine 2000 (Invitrogen) transfection reagents as recommended by the suppliers.

### Plasmid construction

Construction of pGL-U85, pGL-U85CD and pGL-U90 expression plasmids has been described (27,48,50). To generate pTRRAP-mgU2-47, a fragment of human genomic DNA encompassing the second intron and the flanking exons of the transformation/transcription domain-associated protein (TRRAP) gene was polymerase chain reaction (PCR)-amplified and inserted into the HindIII and EcoRI sites of the pcDNA3 expression plasmid (Invitrogen) by using the PCR-introduced HindIII and EcoRI restriction sites. To obtain pGL-mgU2-47, the coding region of the human mgU2-47 gene together with its 93 bp upstream and 99 bp downstream flanking regions was PCR-amplified and inserted into the ClaI and XhoI sites of the pCMV-globin (pGL) expression plasmid (27). Construction of other pGL-based recombinant plasmids designed for transient expression of the U85CD-mgU2-47, U90(hp1-mgU2-47), mgU2-47(hp1-U90) and mgU2-47(tSL-U90) composite RNAs and the mgU2-47-*dhp1*, mgU2-47-*dhp1-dph2*, mgU2-47-*dhp2*, U90-*dhp1*, U90-*dhp3*, U90-*dhp2-dhp3*, mgU2-47-*d1*, mgU2-47-*d2*, mgU2-47-*d3*, mgU2-47-*iL1*, mgU2-47-*iL2*, mgU2-47-*iL1+iL2*, mgU2-47-*tL1*, mgU2-47-*TETR*, mgU2-47-*tS1*, mgU2-47-*tS2*, mgU2-47-*tS3*, mgU2-47-*tS4*, mgU2-47-*tS5* and mgU2-47-*tS6* box C/D RNAs was performed by PCR-amplification approaches using appropriate mutagenic primers. Structures of the expressed mutant U90 and mgU2-47 RNAs are described in the text and/or illustrated in the corresponding figures. The identity of each construct was verified by sequence analysis.

### Immunoprecipitation and RNA analysis

HeLa cells transiently transfected with the appropriate expression plasmids were rinsed with phosphate-buffered saline (PBS) solution, scraped and collected by centrifugation. Cells were resuspended in 1 ml of cold NET-2 buffer (50 mM Tris-HCl pH 7.5, 200 mM NaCl and 0.05% Nonidet P40) and disrupted by sonication with a Bioruptor Plus Sonicator (Diagenode) (five times for 30 s with 30 s intervals at high setting). Cell homogenates were clarified by centrifugation at 16 000 x g for 10 min. For immunoprecipitation, 5 μg of rabbit polyclonal WDR79-C2 antibody (Innovagen) was coupled to 20 μl of packed protein A agarose beads (Sigma) and incubated with 0.5 ml of clarified cell extract for 1 h at 4°C with continuous agitation. Beads were washed four times with NET-2 buffer and used for RNA purification by proteinase K treatment for 30 min at 37°C followed by phenol–chloroform extraction. The purified RNAs were size-fractionated on a 6% denaturing polyacrylamide gel and electroblotted onto a Hybond-N nylon membrane (GE Healthcare). The immobilized RNAs were probed with 5′-terminally labelled sequence-specific oligodeoxynucleotides. RNA structure prediction was performed by using the mfold software (52).

### Fluorescent *in situ* hybridization

Fluorescent *in situ* hybridization experiments were performed essentially as described earlier (27). HeLa cells transfected with the appropriate expression plasmids using Lipofectamine 2000 were plated on 13 mm glass coverslips in 6-well plates and incubated for 24 h. Cells were fixed in PBS supplemented with 4% of paraformaldehyde (PFA) for 20 min, washed three times with PBS and permeabilized with 0.5% Triton X-100 for 4.5 min. The permeabilized cells were washed with 2× SSC (300 mM NaCl, 30 mM sodium citrate, pH 7.0) containing 15% formamide before hybridization with 10–50 ng of fluorescently Cy3-labelled sequence-specific oligodeoxynucleotides at 37°C overnight. Cells were rinsed twice with 2× SSC containing 15% formamide and once with 1× SSC without formamide. For detection of CBs, cells were post-fixed in 4% PFA for 10 min, then washed twice with PBS and incubated with polyclonal rabbit anti-coilin antibody (1:100) (kindly provided by Dr. M. Carmo-Fonseca) for 1 h. After rinsing three times with PBS, cells were incubated with murine IgG1 coupled to fluorescein isothiocyanate (FITC) (1:100) (Sigma). Cells were washed again three times in PBS and stained with 4′,6-diamidino-2-phenylindole (DAPI; 1.25 μg/ml) for 10 s. The coverslips were mounted by Citifluor medium (Citifluor, London, United Kingdom). Chemical conjugation of FluoroLink Cy3 monofunctional dye (GE Healthcare) to amino-modified oligodeoxynucleotides and image acquisition have been described (27). The following modified oligodeoxynucleotide probes were used to detect transiently expressed mgU2-47 (*TCTTATCAGTTTGACTGTCATGGCCT*), U85 (CT*GGGCTTAGCTAAACCAACT*GAATCACAACAGCCTTGAT*A) and U90 (*TACACCCAATTATCTCTATTTCATCATTTCCAT*) RNA sequences. Aminoallyl-modified thymidines are indicated by asterisks.

## RESULTS

### Human mgU2-47 box C/D RNA accumulates in Cajal bodies

**Figure 1.**
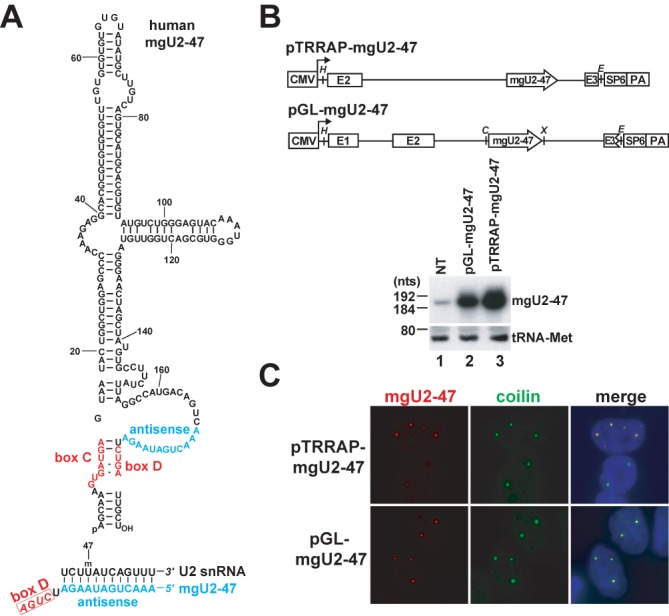
Human mgU2-47 is a novel intron-encoded box C/D scaRNA. (**A**) Primary and predicted secondary structure of mgU2-47. The conserved C and D boxes and the predicted antisense element are highlighted in red and blue, respectively. Potential base-pairing interaction of mgU2-47 and U2 snRNA is shown. The selected 2′-O-methylated nucleotide (U47m) is indicated. (**B**) Transient overexpression of mgU2-47 in HeLa cells. Schematic structures of the pTRRAP-mgU2-47 and pGL-mgU2-47 expression constructs with the cytomegalovirus promoter (CMV), the polyadenylation site (PA), and the exons (E1–E3) of the human TRRAP and β-globin genes are indicated. The relevant restriction sites are shown (*H*, HindIII; *E*, EcoRI; *C*, ClaI; *X*, XhoI). RNAs isolated from HeLa cells non-transfected (NT) or transfected with the indicated expression plasmid were analysed by Northern blotting with a mixture of oligonucleotide probes specific for mgU2-47 and tRNA-Met. (**C**) *In situ* localization of transiently overexpressed mgU2-47 RNAs. HeLa cells transfected with the indicated expression plasmids were probed with a fluorescent oligonucleotide probe complementary to the mgU2-47 scaRNA. Immunofluorescence staining of CBs was performed with an anti-coilin antibody. Nuclei were stained with DAPI.

Identification of *cis*-acting RNA elements targeting vertebrate box C/D 2′-O-methylation guide scaRNAs into the CB has been hindered by the fact that, as compared to the 130–150 nt-long box H/ACA scaRNAs, the known box C/D scaRNAs are notoriously long (330–420 nt) and their secondary structures are poorly defined (27,29). During analysis of HeLa cellular RNAs associated with the WRD79 scaRNP protein (53), we have identified a novel box C/D RNA that was predicted to direct synthesis of the evolutionarily conserved Um47 2′-O-methyl-uridine in the human U2 spliceosomal snRNA (1) (Figure 1A). The new box C/D RNA was termed mgU2-47 (methylation guide for U2 at position 47). During the course of our study, mgU2-47 was also detected by transcriptome-wide analysis of box C/D protein-associated RNAs in human embryonic kidney cells (54).

Northern blot analysis and 5′-end mapping demonstrated that the human mgU2-47 RNA is 189 nt-long (Figure 1B, lane 1 and data not shown). Computer-mediated RNA folding predicted that mgU2-47 folds into a major hairpin structure that carries a short side hairpin (Figure 1A). The genomic copy of mgU2-47 is located in the second intron of the human TRRAP gene in the sense orientation, suggesting that the mgU2-47 RNA is processed from the TRRAP pre-mRNA. To confirm this assumption, the second intron of the human TRRAP gene, together with its flanking exons (E2 and 3), was PCR-amplified and placed behind the cytomegalovirus (CMV) promoter of the pcDNA3 expression vector (Figure 1B). In parallel, the coding region of the mgU2-47 gene was inserted into the pGL expression construct that had been developed for transient expression of intronic RNAs (27,55). The resulting pTRRAP-mgU2-47 and pGL-mgU2-47 expression plasmids were transfected into HeLa cells. Northern blot analysis demonstrated that mgU2-47 was faithfully and efficiently processed from the transiently expressed TRRAP and *β*-globin pre-mRNAs (lanes 2 and 3).

Based on its predicted role in U2 snRNA modification, the mgU2-47 RNA was expected to accumulate in CBs (27). To determine the subcellular localization of mgU2-47, we performed fluorescent *in situ* hybridization (FISH) experiments (Figure 1C). Like most scaRNAs characterized before, the endogenous HeLa mgU2-47 RNA was not detectable with a single oligonucleotide probe in non-transfected cells due to its low level of accumulation (data not shown). However, in cells carrying the pTRRAP-mgU2-47 and pGL-mgU2-47 expression plasmids the ectopically overexpressed mgU2-47 RNA showed strong concentration in 2–8 nucleoplasmic dots. Double staining of the transfected cells with an antibody specific for coilin, a commonly used CB marker protein (56), confirmed that mgU2-47 concentrated in CBs. Thus, we concluded that the newly identified WDR79-associated mgU2-47 RNA is a *bona fide* intron-encoded box C/D scaRNA.

### RNA elements targeting of mgU2-47 into the CB

Identification of mgU2-47, the shortest known human box C/D scaRNA, provided us with an attractive test RNA for experimental dissection of the CB localization element of vertebrate box C/D scaRNAs. Mutant mgU2-47 RNAs and other intronic scaRNAs were transiently expressed in HeLa cells by using the pGL expression plasmid. Accumulation and subnuclear localization of each test RNA was determined by Northern blot and FISH analysis by using radioactively and fluorescently labelled sequence-specific oligonucleotide probes, respectively.

**Figure 2.**
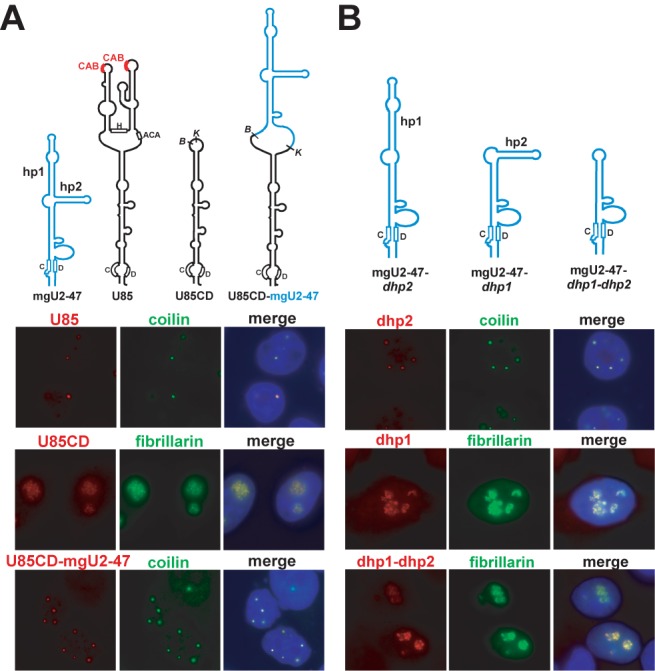
The CB-specific accumulation of human mgU2-47 is supported by the apical hairpin of the RNA. (**A**) The internal part of mgU2-47 carries CB-localization elements. Predicted schematic structures of the human mgU2-47 and U85 wild-type scaRNAs, the U85CD-mgU2-47 composite scaRNA and the U85CD snoRNA are shown. The conserved C and D boxes, the H/ACA-type CB localization elements (CAB boxes) and the apical (hp1) and lateral (hp2) hairpins of mgU2-47 are indicated. Representative images of HeLa cells expressing U85, U85CD and U85CD-mgU2-47 RNAs are shown after hybridization with sequence-specific fluorescent oligonucleotides. CBs were detected with anti-coilin antibody. Nucleoli were visualised by transient expression of GFP-fibrillarin. Nuclei were stained with DAPI. (**B**) *In situ* localization of transiently expressed internally truncated mgU2-47 RNAs. Schematic structures of mutant test RNAs lacking the distal (dhp1, G40-U93), lateral (dhp2, C98-G123) or both (dhp1-dhp2) hairpins of mgU2-47 are shown. Besides massively accumulating in CBs, mgU2-47-*dhp2* was also detected weakly in the nucleoli of about 20% of the transfected cells. For other details, see the legend to panel A.

The human U85 RNA is a composite box C/D-H/ACA scaRNA that is targeted into the CB by two H/ACA-type CAB motifs located in the terminal loops of the H/ACA domain (48) (Figure 2A). Confirming our previous results, the transiently expressed full-length U85 RNA co-localized with coilin in CBs. Upon removal of the H/ACA part of U85, the remaining U85 C/D domain, called U85CD RNA, accumulated in the nucleolus together with the co-expressed GFP-tagged nucleolar maker protein, fibrillarin. We tested whether insertion of the G14-U180 internal sequences of mgU2-47 lacking its terminal C/D core motif into the U85CD nucleolar RNA could restore the CB-specific accumulation of the resulting U85CD-mgU2-47 composite RNA. Northern blot analysis confirmed that the U85CD-mgU2-47 composite RNA efficiently accumulated in transfected cells, demonstrating that the C/D core motif of U85 supported the correct processing and metabolic stability of the large RNA (data not shown). The transiently expressed U85CD-mgU2-47 RNA co-localized with coilin in CBs, indicating that the mgU2-U47 RNA contains internal CB localization signal sequences and/or structures which target U85CD-mgU2-47 into the CB. This observation also excluded the formal possibility that the C/D core motif directs the CB-specific accumulation of mgU2-47, for example through tethering putative box C/D scaRNP protein(s).

To further localize the *cis*-acting CB targeting element of mgU2-47, we expressed internally truncated mgU2-47 RNAs lacking the distal (hp1), side (hp2) or both (hp1-hp2) hairpins of the wild-type RNA (Figure 2B). Removal of the short side hairpin had no major affect on the CB-specific accumulation of the mutant mgU2-47-*dhp2* RNA, but deletion of the distal hairpin resulted in nucleolar accumulation of the mgU2-47-*dhp1* RNA. As expected, the double mutant mgU2-47-*dhp1*-*dhp2* RNA that lacked both hp1 and hp2 accumulated in the nucleoli. These results confirmed that the G40-U93 apical hairpin carries signalling element(s) essential for CB-specific targeting of mgU2-47.

### Human mgU2-47 and U90 scaRNAs possess interchangeable CB localization elements

**Figure 3.**
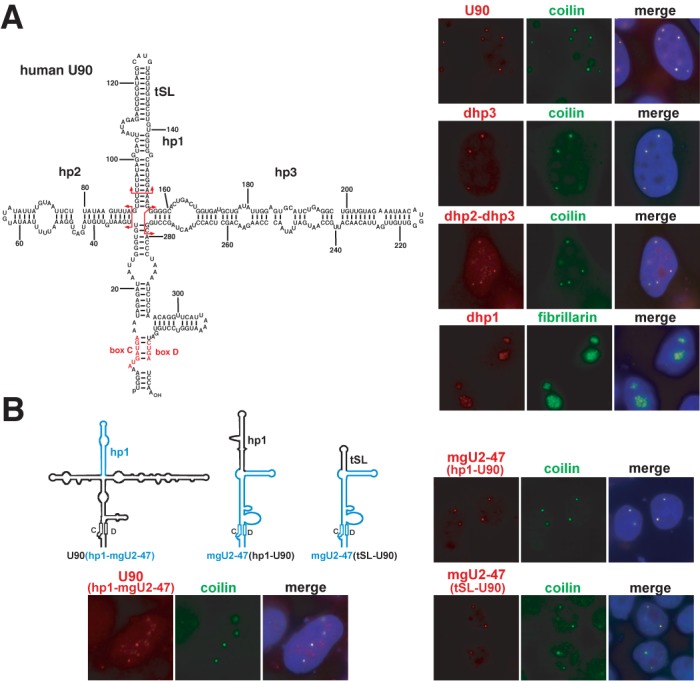
RNA elements targeting the human U90 box C/D scaRNA into the CB. (**A**) *In situ* localization of transiently expressed wild-type and mutant U90 RNAs. Predicted secondary structure of U90 is shown. In the U90-*dhp1*, U90-*dhp3* and U90-*dhp2-dhp3* mutant RNAs, the hp1, hp2 and hp3 hairpins were deleted as indicated by red arrows. (**B**) *In situ* localization of U90 and mgU2-47 hybrid RNAs. Schematic structures of the transiently expressed composite RNAs are shown. The U90 regions are in black and the elements originated from mgU2-47 are in blue.

The human U90 RNA is another intron-encoded box C/D scaRNA implicated in 2′-O-methylation of the U1 snRNA (27). Computer-mediated RNA modelling predicted that the 330 nt-long U90 RNA folds into a cross-shaped overall secondary structure (Figure 3A). Consistent with our previous results, transiently overexpressed wild-type U90 RNA concentrated in CBs (Figure 3A). Deletion of the long right hairpin (hp3) alone or together with the left hairpin (hp2) had no major influence on the CB-specific accumulation of the truncated U90-*dhp3* and U90-*dhp2-dhp3* RNAs, although in about 30% of the cells a small fraction of U90-*dhp2-dhp3* also appeared in the nucleolar structures. Removal of the apical hairpin (hp1) of U90, however, fully abolished the CB-specific accumulation of U90-*dhp1* RNA that was detected exclusively within the nucleoli of transfected cells. This observation indicated that the apical hairpin of U90 carries structural information crucial for CB-specific accumulation of the human U90 scaRNA.

We tested whether the newly identified CB localization hairpins of the mgU2-47 and U90 scaRNAs are functionally interchangeable. The apical hairpins (hp1) of mgU2-47 and U90 were exchanged and the resulting U90(hp1-mgU2-47) and mgU2-47(hp1-U90) hybrid RNAs were expressed in HeLa cells (Figure 3B). FISH demonstrated that the accumulating U90(hp1-mgU2-47) and mgU2-47(hp1-U90) RNAs concentrated predominantly or exclusively in the CBs of transfected cells, respectively. Moreover, another composite RNA, mgU2-47(tSL-U90), in which the apical hairpin of mgU2-47 was replaced with the short G113-U138 terminal stem-loop (tSL) of U90 specifically accumulated in CBs. These results confirmed that the CB localization elements located within the G40-U93 apical hairpin of mgU2-47 and in the G113-U138 terminal stem-loop of U90 are functionally interchangeable.

### The terminal stem-loop of the apical hairpin targets mgU2-47 into the CB

**Figure 4.**
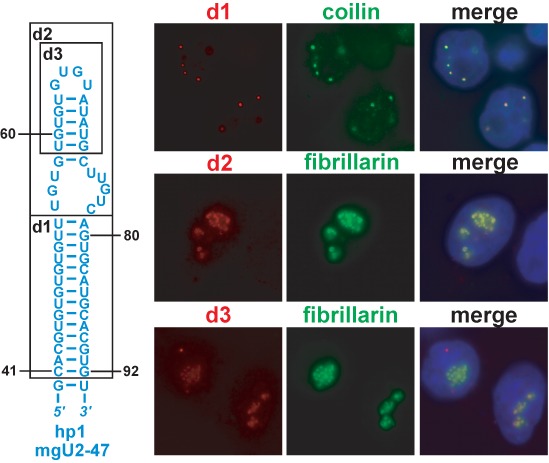
Deletion analysis of mgU2-47. Internally truncated mgU2-47 RNAs were transiently expressed and localized by FISH. Predicted structure of the apical hairpin of mgU2-47 is shown. The deleted d1, d2 and d3 sequences are boxed. For other details, see the legend to Figure 2.

To further delimit the CB localization element of the human mgU2-47 scaRNA, its apical hairpin (hp1) was subjected to deletion analysis (Figure 4). Removal of the d1 (C41-U54/A79-G92) internal segment of hp1 had no effect on the CB-specific localization of the expressed mgU2-47-*d1* RNA. In contrast, upon deletion of the d2 (U55-C78) or the shorter d3 (U59-G72) distal parts of hp1, the truncated mgU2-47-*d2* and mgU2-47-*d3* RNAs accumulated in the nucleoli, demonstrating that the terminal part of the apical hairpin of mgU2-47 carries essential CB localization elements.

**Figure 5.**
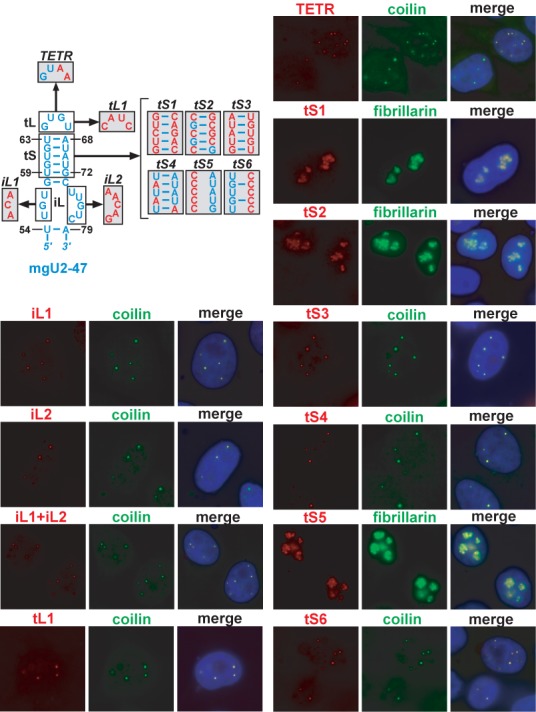
Mutational analysis of mgU2-47. The wild-type mgU2-47 sequences are in blue. Nucleotide alterations introduced into the internal loop (*iL1* and *iL2*), terminal stem (*tS1*-*tS6*) and terminal loop (*tL1, TERT*) of the apical hairpin of mgU2-47 are shown in red. For other details, see the legend to Figure 2.

We performed a detailed mutational analysis of the d2 (U55-C78) terminal hairpin region of mgU2-47 that is composed of a small internal loop (iL), a short terminal stem (tS) and a little terminal loop (tL) (Figure 5). Replacement of the raising U55-U57 or the descending U74-C78 sequences of the iL region with the complementary sequences failed to disrupt the CB-specific accumulation of the mutant mgU2-47-*iL1* and mgU2-47-*iL2* RNAs. Likewise, the mgU2-47-*iL1+iL2* double mutant RNA that featured both the *iL1* and *iL2* base changes localized to CBs. These results demonstrated that the U55-U57/U74-C78 internal loop sequences are not crucial for CB localization and they also suggested that the CB targeting element(s) are confined to the G58-C73 terminal stem-loop of mgU2-47.

Upon random alteration of the G64-U67 terminal loop (tL) sequence, the resulting mgU2-47-*tL1* RNA accumulated in CBs (Figure 5). Likewise, capping the terminal stem (tS) with a GUAA tetraloop that forms a stable tertiary structure and enforces the adjacent RNA helix failed to alter the CB-specific accumulation of the mgU2-47-*TETR* RNA, indicating that the base composition and structure of the terminal loop has no influence on the subnuclear localization of mgU2-47.

**Figure 6.**
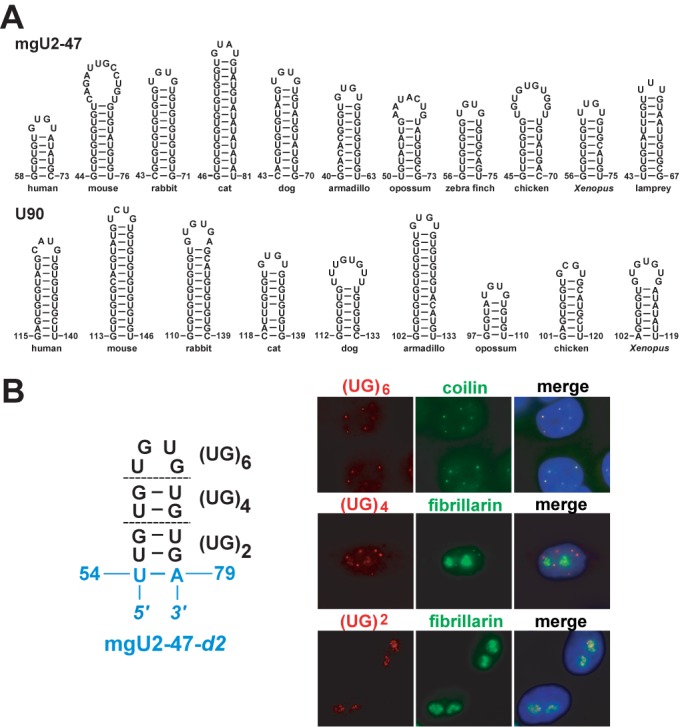
GU-rich repeat sequences target intronic box C/D scaRNAs into the CB. (**A**) Evolutionarily conservation of the CB localization elements of mgU2-47 and U90 box C/D scaRNAs. Vertebrate U90 and mgU2-47 sequences identified by BLAT search (UCSC Genome Browser) were aligned to maximum homology (see Figures S1 and S2). Sequences enriched in GU repeats and overlapping with the experimentally defined CB localization elements (tSL) of the human U90 and mgU2-47 RNAs were folded by the mfold RNA structure prediction software. (**B**) Targeting of the mgU2-47-*d2* nucleolar RNA into the CB by insertion of UG dinucleotide repeats. Nucleotides derived from the truncated mgU2-47-*d2* RNA are in blue. For other details, see the legend to Figure 2.

Comparison of the newly identified G58-C73 CB-localization stem-loop of mgU2-47 with the G113-U138 terminal stem-loop of U90 that successfully restored the CB-specific accumulation of the mgU2-47-*dhp1* nucleolar RNA [see mgU2-47(tSL-U90) in Figure 3B], revealed that the most conspicuous common feature of the two stem-loop structures is that their stems (tS) are built predominantly of regularly alternating G.U and U.G wobble base-pairs with a few G to A substitutions. Mutations which changed the base composition of both strands of the wild-type terminal stem, but sustained the double-stranded nature of the mutant *tS1* helix, fully eliminated the CB-specific localization of the resulting mgU2-47-*tS1* RNA that accumulated in the nucleoli. Stabilization of the terminal stem by systematic conversion of the wild-type G.U, U.G and U:A base-pairs into G:C and C:G pairs fully abrogated the CB-specific localization of mgU2-47-*tS2* RNA. Even more tellingly, swapping the nucleotides between the two sides of the wild-type tS helix of mgU2-47, that altered the nucleotide order in both strands, but did not violate the (R.U/U.R)n principal building concept of the stem, had no effect on the CB-specific accumulation of the mgU2-47-*tS3* RNA. Although A:U and U:A base-pairs are less frequent in the predicted CB localization stem-loops of human and other vertebrate U90 and mgU2-47 scaRNAs (see Figure 6A), the mgU2-47-*tS4* RNA that carried a terminal stem composed of alternating A:U and U:A base-pairs concentrated in CBs. As expected, disruption of the helical structure of mgU2-47 wild-type tS by replacement of the U59-U63 raising sequence with a stretch of five consecutive C residues disrupted the CB-specific accumulation of mgU2-47-*tS5.* On the other hand, substitution of the descending A68-G72 tS sequence with five C residues failed to interfere with the CB-specific localization of mgU2-47-*tS6.* This seemingly contradictory observation, however, could be explained by the fact that the U55-G65 wild-type sequence of the mgU2-47-*tS6* RNA is predicted to form a 4 bp (U.G/U.G)_2_ helix topped with a four nucleotide-long loop, a structure that, based on our results, can likely serve as a CB localization signal (see also below). These results, besides identifying the G58-U63/A68-C73 terminal stem of mgU2-47 as an essential CB localization element, also demonstrated the importance of the (R:U/U:R)n sequence organization of the stem.

### Targeting of a nucleolar box C/D RNA into the CB by insertion of UG dinucleotide repeats

To investigate the evolutionarily conservation of the newly defined CB localization element of intronic box C/D scaRNAs, we identified mgU2-47 and U90 RNA genes in various mammalian, bird, lizard, turtle, frog and/or fish genomes by BLAT search (UCSC Genome Browser). Like their human counterparts, the novel vertebrate mgU2-47 and U90 genes were found within introns of the TRRAP and KPNA4 genes, respectively. Alignment of the inferred RNA sequences showed that apart from the highly conserved box C/D, C′/D′ and antisense sequences, vertebrate mgU2-47 and U90 RNAs show moderate sequence conservation (Supplementary Figures S1 and S2). However, contrary to their weak overall conservation, all mgU2-47 and U90 RNAs carried 20–40 nt-long (GU)- and at much lower extent (AU)-rich internal sequences which were predicted to form the terminal stem-loop of the apical hairpins of the RNAs (Figure 6A). Thus, we concluded that vertebrate mgU2-47 and U90 intron-encoded box C/D scaRNAs share a common CB localization element.

To further corroborate the idea that the evolutionarily conserved (GU)-rich stem-loops target vertebrate intron-encoded box C/D scaRNAs into the CB, we attempted to restore the CB-specific accumulation of the mgU2-47-*d2* nucleolar RNA that lacks its natural CB-localization element by insertion of increasing number of UG dinucleotide repeats (Figure 6B). Insertion of two UG dinucleotides into the top of the truncated apical hairpin of mgU2-47-*d2* failed to restore the CB-specific accumulation of the mgU2-47-*d2-(UG)_2_* RNA that, like mgU2-47-*d2* (see Figure 4), concentrated in the nucleolus (Figure 6B).However, insertion of four UG dinucleotides already partially restored the CB-specific targeting of mgU2-47-*d2-(UG)_4_* RNA that accumulated both in the nucleoli and CBs. Inclusion of six UG repeats was sufficient to target the extended mgU2-47-*d2-(UG)_6_* RNA exclusively into the CBs of transfected cells. As expected, further extension of the inserted UG simple repeat sequence did not affect the CB-specific localization of the resulting RNAs (data not shown). These results demonstrated that correctly positioned UG repeat sequences can target box C/D scaRNAs into the CB.

### CB-specific accumulation of intronic box C/D scaRNAs is accompanied by WDR79 association

**Figure 7.**
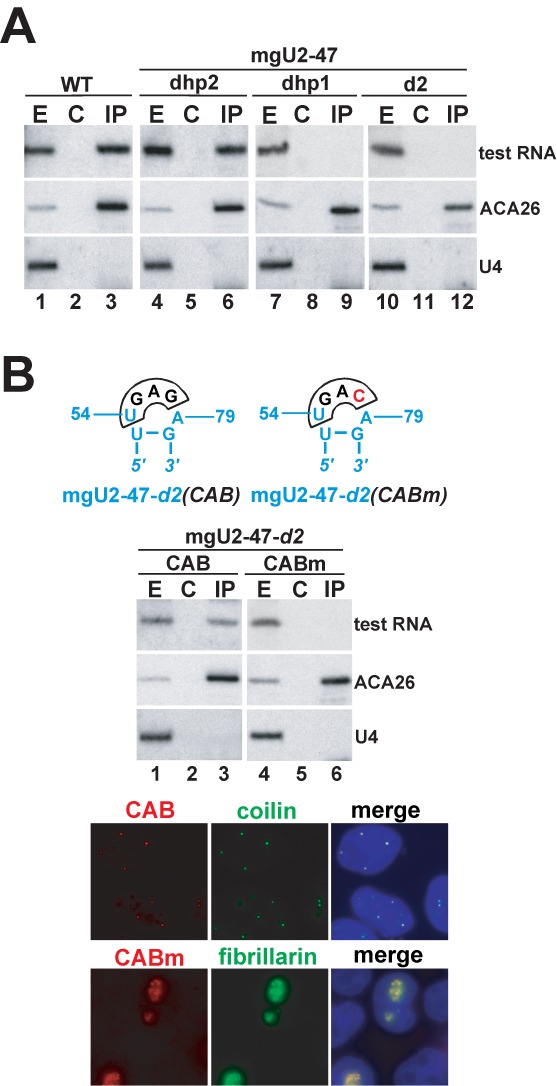
CB-specific accumulation of box C/D scaRNAs requires association with WDR79. (**A**) Co-IP of wild-type and mutant mgU2-47 RNAs with WDR79. Cell extracts prepared from wild-type HeLa cells (WT) or from cells transiently expressing the indicated mgU2-47 RNAs were reacted with an anti-WDR79 antibody (IP). Lane C indicates control IP with no antibody. RNAs prepared from cell extracts (E) or from the beads (IP and C) were analysed by Northern blotting with a mixture of oligonucleotide probes specific for the mgU2-47 test RNAs, the ACA26 box H/ACA scaRNA and the U4 spliceosomal snRNA. About 20 times more cell extract was used for each IP reaction than for input RNA (E) preparation. (**B**) Targeting of the mgU2-47-*d2* nucleolar RNA into the CB by box H/ACA-type CAB box motif. Nucleotides derived from the mgU2-47-*d2* RNA are in blue. The CAB sequences are boxed and the mutant C residue in CABm is highlighted by red. Subnuclear localization of the transiently expressed mgU2-47-*d2(CAB*) and mgU2-47-*d2(CABm)* RNAs was determined by FISH and their interaction with WDR79 was monitored by co-IP experiments followed by Northern blot analysis. For other details, see Figure 2 and panel A.

Human WDR79 has been reported to associate with both box H/ACA and C/D scaRNAs, although it forms a several-fold less-efficient interaction with C/D RNAs (45). The human mgU2-47 box C/D scaRNA has been initially detected amongst RNAs co-purified with WDR79 (see above). IP of HeLa endogenous WDR79 with a specific antibody successfully recovered mgU2-47, confirming its *in vivo* association with WDR79 (Figure 7A, lane 3). During the course of our study, we observed that the WDR79-binding capacity of each mgU2-47-derived test RNA showed a firm positive correlation with its CB-specific localization. In other words, all mutant mgU2-47 RNAs accumulating in CBs were successfully recovered by IP of WDR79 (lanes 3 and 6, and data not shown), but none of the nucleolar mgU2-47 test RNAs showed detectable interaction with WDR79 (lanes 9 and 12, and data not shown).

To confirm the assumption that WDR79 binding promotes the CB-specific accumulation of box C/D scaRNAs, we added an H/ACA-type WDR79-binding motif, the UGAG CAB box consensus, to the top of the truncated hp1 region of the mgU2-47-*d2* nucleolar RNA (Figure 7B). The expressed mgU2-*d2(CAB)* RNA co-purified with HeLa endogenous WDR79 (lane 3) and as demonstrated by FISH, it accumulated in the CBs of transfected cells. The G4 residue in the H/ACA-type CAB box is indispensable for WDR79 binding and CB localization of box H/ACA scaRNAs (45,48,49). Replacement of the G4 residue in the synthetic CAB box of mgU2-47-*d2(CAB)* with a C residue fully abolished both the WDR-binding capacity and CB localization of the resulting mgU2-47-*d2(CABm)* RNA (lane 6). Thus, our results provide further support to the notion that the newly identified (GU)-rich CB localization signal of box C/D intronic scaRNAs, similar to the previously characterized H/ACA CAB box, functions as WDR79-binding site and that binding of WDR79 promotes, directly or indirectly, the CB-specific accumulation of box C/D scaRNAs.

## DISCUSSION

Targeting nuclear RNPs into the appropriate subnuclear compartments where the function is important for accurate eukaryotic gene expression. Both box C/D and H/ACA RNAs are synthesized in the nucleoplasm and after nucleolytic processing and assembly with C/D and H/ACA proteins, the mature RNPs are specifically targeted either into the nucleolus (snoRNPs) or into the CB (scaRNPs), depending on their functions. Previous works demonstrated that the nucleolar accumulation of snoRNPs is supported by the box C/D and H/ACA core motifs, but targeting of scaRNPs into the CB requires additional *cis*-acting RNA elements and at least one CB localization protein, WDR79 (see Introduction). While the CB-targeting element of human box H/ACA scaRNAs, the CAB box, has been extensively characterized, no CB localization signal has been identified for vertebrate box C/D scaRNAs.

In this study, we have demonstrated that the CB-specific accumulation of human mgU2-47 and U90 intron-encoded box C/D scaRNAs is supported by (GU)-dominated dinucleotide repeat sequences which are predicted to form the terminal stem-loop of the apical hairpin of the RNAs (Figures 3–5). We propose that the experimentally defined (GU)-rich terminal stem-loop represents the common *cis*-acting CB localization element of vertebrate intron-encoded box C/D scaRNAs (Figures 6A, S1 and S2). Besides mgU2-47 and U90, human cells also express the mgU2-19/30 intronic box C/D scaRNA that has an unusual structural organization (29). The full-length mgU2-19/30 scaRNA is composed of two tandemly arranged box C/D domains separated by a 175 nt-long spacer. In HeLa cells, the mgU2-19/30 doublet box C/D RNA is partially processed into the mgU2-19 and mgU2–30 box C/D monomers through removal of the spacer sequences. In contrast to the full-length RNA that localizes to the CB, the processed mgU2-19 and mgU2–30 RNAs accumulate in the nucleolus (29). This indicates that the spacer region carries the information required for the CB-specific accumulation of mgU2-19/30. Alignment of vertebrate mgU2-19/30 scaRNA sequences revealed that their spacer sequences are highly enriched in GU dinucleotide repeats, corroborating the notion that (GU)n simple repeat sequences play a crucial role in CB-specific localization of intronic box C/D scaRNAs (Supplementary Figure S3).

The human mgU2-25/61 and mgU12-22/U4-8 box C/D scaRNAs, instead of being processed for pre-mRNA introns, are synthesized from independent genes by RNA Pol II and they carry methylated guanosine caps at their 5′ termini (29). While the 5′-terminal region of vertebrate mgU2–25/61 RNAs is remarkably rich in repeated GU and to less extent AU dinucleotides, the mgU12-22/U4-8 RNAs lack (GU) repeat sequences (Supplementary Figures S4 and S5). The processed 3′-terminal mgU2-61 box C/D monomer of mgU2-25/61 that lacks (GU) dinucleotide repeats concentrates in the nucleolus, further pointing to the importance of GU repeats (29). Unfortunately, removal of the 5′-terminal (GU)-rich sequences of the mgU2-25/61 gene abolished accumulation of the truncated mgU2-25/61 RNA in transiently transfected cells (our unpublished observation). Thus, at the moment it is unclear how Pol II-synthesized, capped box C/D scaRNAs are targeted into the CB. The demonstration that an H/ACA-type CAB box can target a box C/D RNA into the CB raises the possibility that short CAB-like motifs (ugAG) present in many copies in mgU2-25/61 and mgU12-22/U4-8 sequences, may also contribute to the CB-specific accumulation of independently transcribed C/D scaRNAs.

Similar to the H/ACA CAB box, the newly identified C/D-type CB-localization element is not required for efficient accumulation of box C/D scaRNAs, but RNAs lacking a functional CB-localization element accumulate in the nucleolus. On the other hand, however, we failed to target the 60 and 82 nt-long human U75 and mouse MBII-52 canonical box CD snoRNAs into the CB by insertion of the CB-localization stem-loop of mgU2-47 (our unpublished data). The most plausible explanation of this observation is that correct positioning of the CB-targeting element relative to the box C/D core is crucial for WDR79 binding and CB localization. Supporting this hypothesis, changing the distance between the H/ACA core motif and the CAB box through manipulating the hairpin length prevents the CB-specific accumulation of box H/ACA scaRNAs (our unpublished data). It is possible that the extended length of vertebrate box C/D scaRNAs supports correct RNA folding and facilitates WDR79 binding.

Our *in vivo* localization studies confirmed that WDR79 binding is a prerequisite for CB-specific accumulation of intronic box C/D scaRNAs (Figure 7). However, contrary to its apparent importance, WDR79 binds about 20-fold less efficiently to box C/D scaRNPs than to box H/ACA scaRNPs (45) (Figure 7A). In our hands, changing the IP conditions as it had been proposed earlier failed to significantly improve the interaction of WDR79 and box C/D scaRNAs (45) (data not shown). Moreover, the artificial H/ACA CAB box (UGAG) that fully restored the CB-specific accumulation of the truncated mgU2-47-*d2* nucleolar RNA failed to improve the WDR79-binding ability of the mgU2-47-*d2(CAB)* scaRNA, as compared to the wild-type mgU2-47 (Figure 7B). In other words, in context of a box C/D RNP, WDR79 shows equally weak affinity to both box H/ACA- and box C/D-type CB-localization elements. This might indicate that strength of WDR79 interaction with box C/D and H/ACA scaRNPs is determined mainly by its different affinity to the C/D and H/ACA core proteins.

The CB-localization signal sequences dominated by GU dinucleotide repeats are predicted to form the stem-loop topping the apical hairpins of vertebrate mgU2-47 and U90 RNAs (Figure 6A). Unexpectedly, the loop nucleotides do not appear to directly contribute to the CB-targeting function, but the adjacent terminal helix that is composed mostly of regularly alternating G.U and U.G wobble pairs is crucial for both CB localization and WDR79 binding (Figures 5–7). Evolutionarily conserved and functionally important G.U and U.G wobble pairs have been identified in cellular RNAs (reviewed in 57–59). The G.U pairs are stabilized by two hydrogen bonds of unique pattern that provokes an asymmetry in the glycosidic bond angles at G (40°) and at U (65°), distinctive from the symmetric angles (55°) of Watson-Crick pairs. The asymmetry in the G and U glycosidic bond angles is responsible for the nonisostericity with Watson-Crick pairs. The atypical geometry of G.U and U.G wobble pairs creates an irregular hollow surface of the shallow groove of the RNA helix. RNA-protein interactions coordinated by G.U and U.G wobble pairs rely mostly on hydrogen bonds formed between the unpaired exocyclic N2 amino group of guanine on the minor grove side and side chains of Gln, Asp, Asn, Glu and His (reviewed in 57). However, more than two consecutive G.U and/or U.G wobble pairs, especially when G.U pairs are succeeded by U.G pairs, are extremely rare (60), indicating that the newly discovered G.U/U.G wobble CB-localization stem that may contain up to 11 consecutive G.U and U.G pairs (e.g. rabbit) represents an unusual, if not unique, RNA structure (Figure 6A).

Although Drosophila WDR79 has been reported to directly interact with the CB-localization signal sequence of box C/D scaRNAs, it remains possible that mammalian WDR79 recognizes the unusual G.U/U.G stem structure in collaboration with other not yet identified protein factor(s). Thus, understanding of the interaction of WDR79 with the G.U/U.G wobble stems of vertebrate intron-encoded box C/D scaRNAs will be an exciting challenge for the future.

## SUPPLEMENTARY DATA

Supplementary Data are available at NAR Online.

SUPPLEMENTARY DATA
