# Supplementary material for: Targeting vertebrate intron-encoded box C/D 2′-O-methylation guide RNAs into the Cajal body
Source: Nucleic Acids Res. 2014 Apr 20;42(10):6616–29. doi: 10.1093/nar/gku287 (PMC4041459; doi:10.1093/nar/gku287)
Supplement: SUPPLEMENTARY DATA [file supp_42_10_6616__index.html]

Targeting vertebrate intron-encoded box C/D 2′-O-methylation guide RNAs into the Cajal body — Targeting vertebrate intron-encoded box C/D 2′-O-methylation guide RNAs into the Cajal body — SUPPLEMENTARY DATA 

# Targeting vertebrate intron-encoded box C/D 2′-O-methylation guide RNAs into the Cajal body

## SUPPLEMENTARY DATA

**Files in this Data Supplement:**

- SUPPLEMENTARY DATA
