# Supplementary material for: Targeting vertebrate intron-encoded box C/D 2′-O-methylation guide RNAs into the Cajal body
Source: Nucleic Acids Res. 2014 Apr 20;42(10):6616–29. doi: 10.1093/nar/gku287 (PMC4041459; doi:10.1093/nar/gku287)
Supplement: SUPPLEMENTARY DATA [file supp_gku287_nar-00609-a-2014-File009.pdf]

SUPPLEMENTARY INFORMATION

Targeting mammalian intron-encoded box C/D 2'-O-methylation guide RNAs into the Cajal body

Aline Marnef, Patrica Richard, Natalia Pinzón and Tamás Kiss

Results

To identify evolutionarily conserved and therefore, functionally important sequences in vertebrate box C/D scaRNAs, the coding genes of the intron-encoded mgU2-47, U90, mgU2-19/30, and the independently transcribed mgU2-25/61 and mgU12-22/U4-8 scaRNAs were identified in mammalian, bird, lizard, turtle, frog and/or fish genomes by BLAT search (UCSC Genome Browser). The predicted RNA sequences were aligned to maximum homology by using the ClustalW2 algorithm developed for multiple sequence alignment (EMBL-EBI). In addition to the conserved C/D and C'/D' boxes and the RNA-specific target recognition sequences, we detected long GU- and AU-rich internal repeat sequences in the intronic mgU2-47, U90 and mgU2-19/30 scaRNAs (Figures S1, S2 and S3). The 5'-terminal regions of the independently transcribed mgU2-25/61 scaRNAs are also enriched in GU dinucleotide repeat sequences (Figure S4). In contrast, compilation and comparison of the Pol II-transcribed mgU12-22-U4-8 box C/D scaRNA sequences failed to identify GU-rich internal sequences (Figure S5).

```
                     box C                                                       tSL
cat           AGCAAAGTGATGAG-CGTTGCTGGCTGGAG-CCCCAAAG----AGGCACACG-TGTGTGTGTGTGTGTGTATGTATGTATATATATATCTGTGCCCTTGTCTGTGCATGCACACGTATGT 113
armadillo     AGCAAAGTGATGAG-CTTTACTGGCTGGAG-CCCCAAAG----AGGCACACG-TGTGTGTGTGTGTGTGTGTCTGTGTTTGTATATATGTATGCTTGTTGTAT-----GCACAGGTGTTT 108
dog           AGCAAAGTGATGAG-CATTATTGGCTAGAG-CCCCAAAG----AGGCACATG-TGTGTATGTGTGTGTATGTATGTGTGTCTGTATCCTTGTCTGTGCATG--------CACATGTATGT 105
human         AGCAAAGTGATGAG-TAATACTGGCTGGAG-CCCCAAAG----AGGCACGTG-TGTGTGTTTGTGTGTGTGTGTATATGCTTGTCAGTGCATGCACGTGTATGT---------------- 97
rabbit        AGCAAAATGATGAG-CCACACTGGCTGGAT-CCCCAAAG----AGGCACGTG-TGTGTGTGTGTGTGTGTGTGTGTGTGTCTGTCCGTGGCCGTCCTTGTCTGTGCGCTCCC-CGTGTGT 112
mouse         AGCACAATGATGAA-CAATACTG-CTGGAG-CCACAGAG----AGGCACAAG-TGTGTGTGCCAGATTGCCTGTGTGTATGTGTGTCCTTGTCTGTGCATG--------CACACGTGAGT 104
opossum       AGCAAAATGATGGA-TATTATTGGTTGGAG-CCCTAAAG----AGGCA-ATA-TGAATGTATATGAATGACTGTATGTG----------------------------CGCGCACTTGTGC 84
chicken       AGCAAAGTGATGAA-TAATATTTCCTTGAG-CCAAAAAG----AGAGAGAGT-TGTGTGTGTGTGTGGTTGTATGA-------------------------------------------- 58
zebrafinch    AGCAAAATGATGAG-AAACACTTGCATGAG-CCGAAGTG----AGTGAGTTG-TGTGTGTGTGTGCAGTGGTGTGTGGTTGTTTGA---------------------------------- 67
Xenopus       GGCAAAATGATGAG-ATCATCAGACTTGACTCCCCACAG----TGTGACTCG-TG-GCACTTGTGTGTGTTTGTGTGCATGTTTGTGCATGT---------------------------- 71
lamprey       AGCAGGATGATGAAAAACTTTTTTCTTGAG-CCCAAAAATAAAATTCACACGTTGTGTATTTGTTT-TGTAATTGTGCGTA----------------------------ACACGTATTC 89

                                                                                antisense    box D
cat           CTGGGAGTTTGAGTGTATGTGTGACTGGTGGTAGGGAACAAGTTAC---GTGCCTTC-TGTT---GGACCATGACAGTCAAACTGATAAGATCTGATTGCT 207
armadillo     CTGGGAGTACAAATG--TGTGTGACTGATTGTAGGGAAATACTTAAT--GTGCCTTC-TGTT---GGACTGTGACAGTCAAACTGATAAGATCTGATTGCT 201
dog           CTGGGAGTACGAATG--TGTCTGACTGATTATAGGGAACTAGTCTAT--GTGCCTTC-TGTT---GGACGATGACAGTCAAACTGATAAGATCTGATTGCT 198
human         CTGGGAGTACAAATG--GGTGCGACTGGTTGTAGGGAACTAGCTAT---GTGCCTTC-TATT---AGGCCATGACAGTCAAACTGATAAGATCTGATTGCT 189
rabbit        CTGG--CTATGCGTG----TGTGTCTGGTTATAGGGAACTAGCTGT---GTGCCTTC-TGTT---GGGCCATGACAGTCAAACTGATAAGATCTGATTGCT 200
mouse         CTGGGAGGATGGATG--TGTGTGACTGGTGGTAGGGACCTAGCCCT---GTGCCATC-TTCT---GGGCTGTGACAGTCAAACTGATAAGATCTGATTGCT 196
opossum       CTGGATGTATGACTG-GAGTATGAATGAGGATAAGGACCTAGTTAT---TTGCCTTT-TTTTATAGGGCCATGATATTCAAACTGATAAGATCTGATTGCT 180
chicken       ------------CAG-------GGGTGTTTGTGAG-----AATTGT---GTGCCTTA-TAAT--TTGGTGGTGACAGCTAAACTGATAAGATCTGATTGCT 140
zebrafinch    ------------GAG-------AACTGT---------------------GTGTCTTA-TAAT--TGGGCAGTGATAGCTAAACTGATAAGATCTGATTGCT 137
Xenopus       ------------------GGGTGACTGGTTGAGTG----TGAGTGAG--CACCTTTC-ACATG--GGTTCCTGAGAGCTAAACTGATAAGATCTGATTGCT 159
lamprey       ACGTTTGTTCATGTA---CACGTGTTGTTTATATGGTTCTGTTTTAATGTTGCACACGTGTGAACGGGCAGTGGAAGGAAAACTGATAAGATCTGACTGCT 187
```

Figure S1. Sequence alignment of vertebrate mgU2-47 RNAs. The conserved box C and D motifs and the antisense sequences are indicated in red and blue respectively. The RU-rich internal sequences are highlighted in yellow. The experimentally defined CB-localization stem-loop (tSL) of human mgU2-47 is shaded in blue.

```
                      box C
human        TGGAAATGATGAAATAGAGATAATTGGGTG-----TTGAATGTTG-TATGACTGGAAATTTTTAATATGTATATTATGTAATTCTTATAAGTTTAGTGTTTTATAGGTACTTAATAG--- 111
rabbit       TGGAAATGATGAAATAGAGATAATTGGGTG-----TTGAATGTTG-TATGACTGGAAATTATTAGTATTAATGTTAAGAAAT---TATAAGTTTAATGTTTTATGGATACTTACTAG--- 108
mouse        TGGAAATGATGAAATAGAGATATTTGGGTG-----TTGAATGTTGCTGTGAGTGGAAAATTTTAATACATGT----AGTAATTC-TCCTAGTTTAATGTTTCATTGATAGTTACTAGTGT 110
dog          TGGAAATGATGAAATAGAGATAATTGGGTG-----TTGAGTGTTG-TGTGATTGGAAAATTTTAACATGTGAGTTAAGTAAT-----TAAGCTTAATATTTTATAGATACTTAATAG--- 106
cat          TGGAAATGATGAAATAGAGATAATTGGGTG-----TTGAATGTTG-TGTGATTGGAAACTTTTCATATGTAAGTTAAGTAAT-----TAAGCTTAATATTTTATAGATAATAGAGTG--- 106
armadillo    TGG-AATGATGAAATAGAGATAATTGGGTG-----TTGAGTGTTG-TATGATTTGAAATTATTAATATTTGT--TATGTAATTAGTATAAGTTTAAAATTTTCTG------TAAT----- 102
opossum      TGGAAATGATGAAACAGAGATAACAGGGTGCA--TTTGAATGGTG-TGTGATTGGAGCATTTTAAAATTTAA--------AT-----TAAGTAAATATTAAAATGGG-----GGGGG--- 96
Xenopus      TGGAAATGATGAAGTTGAGAT-TTTGGGTG-----TTGAGTGAAT-GTTAATTTTAGAATGTGTAAATTTAG--------TT-----GATGTAGTATAACTCGTAG------TAATG--- 91
Chicken      TGGAAGTGATGAAATGGAGATAATTGGATGTCTCTTTGAATGGAG-TGTGTGTGAGGGACAGTCAAATGGGG---------TG----GAAGGATGGGGGGGGTGGG-----GGTGG--- 98

                                        tSL                                                                 antisense    box D'
human        ---------AGAGTGTGTATGCATGTGTGTGTGCTTGTGGTGGCTATG--------------GAAAGGGGGCACTGACTG--GTGATGCTGATATTGGAGTGCATCTGAGGCT-GTTGTA 205
rabbit       -AGTGTGTGTGTGTGTGTGAGCATGTGTGTGCTTATGGTGGCTATG----------------GAAAGGGGGCACTGAGTG--GTGATGCTGATATTGGAGTGCATCTGAGGCT-GTTGTA 208
mouse        AAGTGTGTGCATGTATGTCTGTGTGTGTGTGTGTGTGTGTGTGTGGTGGCTAT-------GAAAGGGGGCACTGAATG--GTGATGCTGATATTGGAGTGCATCTGAGGCT-GTTGTA 220
dog          -----------AATGTGTGTGTGTTTGTGTTTGTGTGCTCATGGAGGCTATT----------G-AAGGGGGCACTGACTG--GTGATGCTGATATTGGAGTGCATCTGAGGCT-GTTGTA 201
cat          -TGTGTGTGTGCCATTTGTGTGTGTGTGTGTGTGTGTGCGTGCACTCATGGAGGCTA---TTGAAAGGGGGCACTGACTG--GTGATGCTGATATTGGAGTGCATCTGAGGCA-GTTGTA 219
armadillo    -------------TGTGTGTGTGTGTGTGTGTGTGTGTGTACATGTGGAGGCTATG------GAAAGGGGGCACTGACAG--GTGATGCTGATATTGGAGTGCATCTGAGGCT-GTTGTA 198
opossum      --------------GTGTATGTGTGTGTGCGTGGGTTTAAGTATAAAGGCTAAG--------GAAAGGAGCCCTGACAG--ATAATGCAGATATCGGAGTGCATCTGAGGCG-GTTGTA 191
Xenopus      --AA------------GTGTGTGTGTGTG-ATATATTTGGTTAT----------------TTGCAAGGGGGCTCTGAAATT-ACATTGCTGATTTCGGAGTGCATCTGAGG---GATGTT 176
Chicken      --GGGA------------GTGTGTGCGTGCATGCTTGTATA----------GAAAAGGCTTTAAATGGGGGCACTGACAATGAAAGTGCTGATTCCGGAGTGCATCTGAGGCATGTTGTT 194

                                                   box C'
human        GAAATAACATGGGTTGTTGATTACAACATTGCCAATGATTATAACCCAAGAACAGCTCACCTA-ACTAGCCTGG-CACCCTAAAATCTCTAACAGGTTCAT---TAAA-ATGGTCCTGTG 319
rabbit       GAAATAACATGGGTTGTTGATTACAACATTGCCGATGATTATGACCCAAAAACAGCTCACCTA-AGTAGCCTAA-CACCCTAAAATCTCTGACAGGTTCAT---TAAACATGGTCCTGTG 323
mouse        GAAATAACATGGGTTGTTGATAACAACGTTGCCGATGATTAAAACCCAAAAACAGCTCACCTA-AGTAGCCCAG-CACCCTAAACTCTCTAACAGGATTAT---TAAACAT---CCTGTG 332
dog          GAAAT-ACACGGGTTGTTGATTACAACGTTGCCGATGATTATAACCCAAGAACAGCTCACCTA-AGTAGCCTAG-CACCCTAAAATCTCTAACAGGTTCAT---TAAACATGGTCCTGTG 315
cat          AAAAT-ACATGGGTTGTTGATTACAACGTTGCCGATGATTATAACCCAAGAACAGCTCACCTA-AGTAGCCTAG-CACCCTAAAATCTCTAACAGGTTCAT---CAAACATGGCCCTGTG 333
armadillo    GAAAT-ACATGGG-TGTTGATTACAACATTGCCTGTGATTATAACCCAAGAACAGCTCACCTA-ACAAGCCTAG-CACCCTAAAATCTCTAACAGGTTCAC---TAAACCTGGTCCTGTG 311
opossum      AAAAT-ACAAGAG-TGTTTATTACAACTTTGCCAGTGATTACAATTTGGAAACGGCTCACCTA-CCTG-CCTGA-CACCCTATAATCTCTAACAGGTTCA----TAAATGTGGACCTGTA 302
Xenopus      GCAATGTGAAGGG--CTTCTCTGCAACATTACCAGTGATTAGAGCCCAT-ATTGGCTCCCTTA-AATAACCTGCACATCCTTATATCTCTAACGGGTGCTTGA-TTAACATGTACCCGTG 291
Chicken      TTAAATTCTGGGGTCCTACTTTCCAACATTGCCAATGATTAAAGCCCAAGACAAGCTCACCAAGACAAGCCTAG-GCATCTTAAATCTCTAACAGGTTCGTTAATAAAAATGATCCTGTG 313

               box D
human        ATCTGATCCAA 330
rabbit       ATCTGATCCAA 334
mouse        ATCTGATCCAA 343
dog          ATCTGATCCAA 326
cat          ATCTGATCCAA 344
armadillo    ATCTGATCCAG 322
opossumU90   ATCTGATCCAT 314
Xenopus      ATCTGATTCAA 302
chickenU90   ATCTGATCCAA 324
             ******* **
```

Figure S2. Sequence alignment of vertebrate U90 RNAs. The conserved 5'-terminal C and 3'-terminal D boxes and the putative internal box C' and D' motifs are indicated in red. The predicted antisense sequences are in blue. The RU-rich internal sequences are highlighted in yellow. The experimentally defined CB-localization stem-loop (tSL) of human U90 is shaded in blue.

```
                                                    box C     antisense box D'                              box C'
chicken      TCCTCTGAGATCTGCAGTTCTACTGAAGTGGGTCTGTGATGAGAAATAGCCAAATCTGAGCACCAG--AAGATAAAT--AATCTTCGAGGTGTCATGATTT------------------- 97
zebrafinch   CCCTCTGAGATCCACAATTCAACTTAAGTGGGTCTGTGATGAGAAATAGCCAAATCTGACCACCAG--AAGATAAAAC-AATCTTCCAGGTGTCATGATTT------------------- 98
cat          CTCTCTGAGATTTGCT--CTTACTGAAGTGAATCAATGATGA-AAATAGACAATTCTGAGCATCAG--AAGACCTTCT-AATTT-GCCTGATGCATGATCC------------------- 94
dog          TTCTCTGAGATCTGCT--CTTACTGTAGTGGATCAATGATGA-AAATAGCCAAATCTGAGCATCAG--AAGACCTTCC-AGTCT-ACCTGATGCATGATCT------------------- 94
human        CTTTCTGAGATCTGCT--TTTAGTGAAGTGGATCAATGATGA-AACTAGCCAAATCTGAGCATCAG--AAGTCTTTCC-AGTCT-ACCTGATGCATGATCT------------------- 94
rabbit       TTCTCTGAGATCTGCT--CATACCAAGGTGGGTCAATGACGA-AAATAGCCAAATCTGAGCATCAG--AAGACCTTCT-AGTCTTACCTGATGCATGATCT------------------- 95
armadillo    TTATCTGAGATCTGCTC-CTAACTGAAGTGGATCAATGATGA-AAATAGCCAAATCTGAGCATCAG--GAGACCTTCT-TGTCC-CCCTGATGCATGATCT------------------- 95
mouse        AAGACTGTTCTCTGAGA-TCTACTGAAATAGATCAGTGATGA-AAATAGCCAAATCTGAGCATCAG--AAGGCCTTTT-GGTCT-----GGGGCATGATCT------------------- 91
lizard       TCCTCTGAGAGTTGCT----TACT-CAGTGGCTCTGTGATGAGAAATAGCCAAATCTGAGCACTTGAAAAGGTCAGCT-GATCT--TCTAGTGCATGATTT------------------- 93
fugu         ATGGCTGCTCCCCTCTGAGGTTCACTCATGAATCAATGATGAAAAATAGCCAAATCTGAGTTCCTGGGCAGTTCTCAGTCCTCTATGATTATCCCTGAGAAGCTGTGTCTGTGTGTGTCT 120
                 ***  .    .     ::     .*.. **:.*** ** **.***.***:*****  :  :*   **   :      *          *.***
                                  box D
chicken      ----------GTATGCAGTTCTGAGAGGCTTCTTTGTGTGTCTG----------------------------------------------------------------------------
zebrafinch   ----------ATATGCAGTTCTGAGAGGCTT-------TATATG----------------------------------------------------------------------------
cat          ----------CTAT--AGTTCTGAAAAGCAAAAAAAAAAAAAAAAAAGTAA-----------TTTAGAAAAATAAGGGCT------------------------------TACGT------ 155
dog          ----------CTCT--AGTTCTGAGAAGCAAAAGTAATTTAGAACAATAAGGGCTTATGGCTGGAGGTGTGTATGTGCG------------------------------TACGTGCATGC 172
human        ----------CTAC--AGTTCTGAGAAGCAAAACTATAAAACAA-----------------TGTAAAACAATAAGGGCA------------------------------TATGT------ 149
rabbit       ----------CCAT--TGTTCTGAGAAGCAAAACTATCATTATT-------------------TAAAACAATATGGGCAT-----------------------------TACATCTG--- 152
armadillo    ----------CTAT--AGTTCTGAGAAGCAGTAGTTTGAAAGGT----------------------------------------------------------------------------
mouse        ----------CTCT--AGTTCTGAGAAGCAAAACTACAAAGCGG-----------------------------------------------------------------TTTGT------ 128
lizard       ----------ATATACTGTTCTGGGAAGCAATTCAAATCTGACG----------------------------------------------------------------------------
fugu         GTGTGTGTCTGTGTGTGGGTGTGTGTGTCTGTCCCTTCCCCTGTGTGTCCGTGTGTGTGTCTGTCCCTTCCTGTGTGTGTGTCCAGCTCTGCGTGTGTCTGTGGGTCTCTCTCTCTCC 240
                    *         * * **  .:.  *:

chicken      -----TATGTGTGTGG-TGTGTGTGTG----TGTGTGTTG-----------------GGGGGAGGGGGCTGCTGGAGGGAATTG----------------TGTCTG--GGAGACTGG--- 203
zebrafinch   -----AGAGTGAGTG--TGTGTATGTG----TGTGTGTAT-----------------GAGG---GGGGCTGTTGG-GGGTGATG----------------TGCCTG--GGACATGGG--- 192
cat          ---CTGGAGTGTGTGTGTGTGTGTGTG----TGTGTGTGT------------ATAAAGATGACAGTGTATAACTGAGGACTTGA----------------TGAATG--AGATTGGGG-TG 237
dog          GTGCGTGTGTGTGTGTGTGTGTGTGTG----TGTGTGTGTGTGTGTATGTGTTTAAAGGTGACCGTGTATAACTGAGGACTTGA----------------TGAATG--AGATTGGG---G 267
human        ---CTGGTGTGTGTGTGTGTGTGTGTG----TGTGTGTGTGTGTGTACG--CACATGTGTTTATAAAGATAACAGCTGTAGGAA----------------TGAATG--AGATTGAGG-GT 241
rabbit       GGGGTTGTGTGTGTGTATGTGTGTATG----TATGTATGTATGCATGTGTCTATAAAGATGGCAGTGGATAACTGAGGACTAGA----------------TGGATG--AAATTGTGTGTG 250
armadillo    -----GTTGTGCGTGCTTGTGGGCACA----TGTATGTGT-----------------GATAACTGAG-ATTGCT----AGTGGG----------------TGAAGG--AGATTGGGGGTG 198
mouse        ---CTGTGGTGAGAGAAGGAAGAGTGG----TGTATGGATG----------------GATGGATGAGGGTGTGT---ACATATC----------------TGTGTG--TGTGTGTGTGTG 204
lizard       --GAGAGCGTGAGTTTTTTTTGGTGCAT---TGTATAAGTG------------GTGAGTGACTGGGAAGAATTTGGGTAGATG----------------AATCGGGCAAAGTGGGGGAG 213
fugu         CCACCTGTGTGTGTGTGTGTGTGTCCGTCCGTGTGTGTGTGTCTCTCCCCACCTCTGCGTGTGTGTGTGTGTCCGTGTGTGTGTCTCTCTCCCACCTCTGTGTG--TGTGTGTGTGTG 358
                     *** *:      :   .    .    *.*.*.                        .      . .                :.    *    .: :  *
                                                                                    box C               box D'                box C'
chicken      -ATGTG----AATGTG---------------------------------------TACTGAGCAATGATGAGTTTA-ATTTG-CCTGACT-----TCAAAAA-------GTGAAGG--CT 263
zebrafinch   -GTGTG----AGTGTGT------------------------------------TGTACTGAGCAAGGATGAATCTATATTTG-CCTGATTG---TGCAAAAC-------ATGAAGG--TT 258
cat          AGTATG---GAGGGGAT--------ATGAAAGA--------------CTAATCATTTCTGAGCAATGATGGAAAGATTTTACTACTGACAT---TT-ACAACT------ATGAATG--TT 320
dog          GGTGGG---GAGGGAAT--------CTGTGTG----------------TAG--ATTTCTGAGCAATGATGAAAAGGTTTTACTACTGATCT---TT-ACAACT------ATGAAGG--TT 346
human        GGGGGG---GTGCGTATGTATGTCTATGAAAGC--------------CTAATCATTTCTGGGCAATGATGAAAAGGTTTTACTACTGATCT---TT-GTAACT------ATGATGG--TT 332
rabbit       TGTGTGTGTGTGTGTGTGTGTGAGTGTGAAAGAGACGGAGAGAGAGGCTAATCTTTTCTGAGCAATGATGAAAAGGTTTTACAACTGACCT---TT-ATAACT------ATGAGGT--TT 358
armadillo    TATGTGAGTGTGAGTGTGTG----TGTGAAAGG----------------CAAACAGTTCTGAGCAATGATGAGAAGGTTTCATGACTGACCT---TTTATAATT------ATGAAGG--CT 288
mouse        TGTGTG--TGTGTGTGT--------GTGTGTGG------------------TCATCTCTGAGCAATGATGAAAAGGTTTTAGTACTGAATT---TT-GTCACT------GTGAAGG--CT 284
lizard       TGTGTGTGTGTGTGTATGCATGTCAGAGAGTGAG--------------AGAAAAGTACTGAGCAGTGATGAACCTA-AGTGGAACTGACAA---TTTAATAC-------ATGGAGG--TT 306
fugu         TGTGTGTCCGTGTGTGTGTCTCTCTCTCTCTCTCTG-------TCTCCACCTCTGCTGAGCAATGATGTGAAATGACACGTCCAGAAATGTATTGAATCAAATGCATTTGATGATGTT 471
              . . *    :. * .                                           ***.***. **** .     :     .*:**         .   .  .**         *
              antisense box D
chicken      TCTACACTTGAACTGAGCTCA 284
zebrafinch   CCTACACTTGAACTGAGCTCA 279
cat          TCTACACTTGATCTGAGCTCA 341
dog          TCTACACTTGATCTGAGCTCA 367
human        TCTACACTTGACCTGAGCTCA 353
rabbit       TCTACACTTGACCTGAGCTCA 379
armadillo    TCTACACTTGACCTGAGCTCA 309
mouse        CCTACACTTGACCTGAGCTCA 305
lizard       TCTACACTTGACCTGAGCTCA 327
fugu         TCTACACTTGAACTGAGCTCA 492
              ********** *********
```

Figure S3. Alignment of vertebrate mgU2-19/30 RNAs. Details are identical to Figure S2.

```
rabbit      ---GTTTAAAAGAGAGTGCGGCCTTCCGCTGCGGCACGGGAAG-AGAGCGGCCTTGGGTGAGGCTTGTGTCGGCTGTGCATGTGTGT---------------------CGCGTGGGAGGC 95
armadillo   ---GTTTAAAAGCGAGCGCGGACTTCCGTTTCTTTAGGGGAGGGAGCGCGGCCTGGGGTTCGGCT-GTGTCTGTGGGGCG---GTGT---------------------CGCGTGGAAGGT 92
human       ---GTTTA-----------------------------GGGAGGGAGAGCGGCCTGGGTCCTGGGTGTTGTGTGCGGAGCTGTGGCGT---------------------CGCGTGTGAGGC 68
cat         --ATTTAAAAGAGG-ATGCGGACTTCCGTTCTCTTTCCGGAGGGAGAGCGGCCTAGGGCG-AGGTGGTTTGTGTGGTGCTTTGGTGT---------------------TGAATGGAAGGC 95
dog         GCCTTTAAAAGGGGGGTGCCGACTTCCGG-CCCTTCCCGGAGGGAGAGCGGCCTGGGGCGGCGGTGCTGTGTGTGGTGCTGCGATGTGCGTGGAAGGCGCGTGGAAGGCGCGTGGAAGGC 119
mouse       ---GTTTAAAAGAGGGTGCC-CCAGCCACTTCCTCGCGAGTGGGAGTGCGG-CTGGGGCTGTGGATGTCTGTGTG-TGGTATTGTGT---------------------GACGTGAATGGT 93
opossum     ---GTTTGGGCAGGGGCGCGGG-TTCTGCATACCGGTTGCTTGGTGTGCG---TGAGGCGGCCGAGTTACGTGCGGAAGGA---------------------------CGCGTGGAAGGC 86
Xenopus     ---GCAATAG-------------------------AACAGTAGGAACAGCG----GCGGCATGGAAATGTATGGCTGTAAT----------------------------GTGAGGTTTGG 61
Tetraodon   ---GCTGTGAGCAAAGAGAG-CCCCCTTTCATTTCCCACAATTCCACGCGGCACTGCGTTTATATTCCGGAAGTTGGTTTGTTTGTC-------------------ACAGTTTGAATGGT 97
fugu        ---GCTGTGAGGAAAGTGC--CCCCTTTTCACCTCCCATGATTCCCTGCGGCTCAGCACTTATACACCGGATGTGGGCTTTTCT----------------------TCAGTTTGAATGGT 93
                 :   .                               :       ***                        *                             .   :*   :  *

rabbit      GTGTGTGGGCCGAG-GCACGAGTGGATG-----CGTGGATGTGTGTGTG-TGTGCGCGCGTGTTGCGTGCAAG-GCGAGTG-AGTGCGC--CCACCCCTGTGCCCCTCTCCCCTCCTTAC 204
armadillo   GCATGTGAGCCGAGTGCGTGAGCGGACG-----CGTG------TGTATG-TGTGCTTGGG----GCGTGCAAG-GCGAGTG-CGTGCGC--CTCCTCCTGCGCCCCTCCCTCCCGCCTAC 192
human       GCGTGCAGGGTGAG--TGTGAGTGGACG-----CGTG--AGTGTGTGAG-TGTGCGCGCTTGGAGCGTGTTAG-GCGAGTG-CGTGCGC--CCACCCCTGCGCCCCTC-CTCCCGCTTAC 173
cat         GCGTATGGGCTGAG--TGTGAGTGGGTG-----CGCG------ACTGTG-AGTGTGCGCGTGGGGCATGCAAG-GCGAGTG-TGTGCGC--CCGTCCCTGAGCCACTCTCCCCCGCCTAC 197
dog         GCGTGTGGGCTGAG--T--GAGTGGGCG-----CGCG------AGTGTGGTGGGTCCACGTGGGGCGTGCAAG-GCGAGCG-TGTGTGCG-CCGTCCCTGAGCCCCTC-CCCCCGTCTAC 220
mouse       GCCTTTGGGCTGAATGCGAGTATGGATA-----TTGG----AGTGTGTG-TGCGAGCGACTGGGAGGTGCAAG-GCGAGTG-TGTGTAC---CCCCCCT---CCCCTC-CCCTCGCCTAC 194
opossum     GATTGAGTG-TGTG--TGTGAGTGCAAGG----CCAG----AGTGCCAG-TGTGTGTGCAAGGCGAGCGCATGTCCGTGTGCCGAGCCGG-CCCTCCCTAAACCTCTC--CCCCCCCTAC 191
Xenopus     TATGAAAGCCTGCTAGTGGGAGTGAATG-----CGAG------TGTGAG---TGGGAGTTGTGCGGCTGTGAGTGTGGAGTTGTGCGCTGCTGCTCCTGCTTTTCTA---------TAC 158
Tetraodon   TTCTTGGAG-TGTGTGTCTGCGTGTTTG-----GGTGAACGAACGAGTG-AGTGTTTGTGT-------GCGCGTTTGTCTG-TCTTTTTT-ATTTTCT-----TTTGTAAAAAAACCTAC 196
fugu        TTCTCCGAG-TGTGTGTGTGAGTGTCTGTCTGCCATGTATGTTGGAGTG-AGTGATTGTGTGAAAGTAGCGTGTGTGTGTG-TGTGTGTG-TGTGTCTACCTATATATATAAAAACCTAC 209
                 .    *       * . *    .        *          :*    *    .         *   *   *   *   :           *                ***

              antisense  box D'                                     box C'
rabbit      ACCTTGATCTTATTTGATCGGGTCGTG-ACCCCAGCCTCGCCGGGCCGTCCCGAAATGAAAAGCTCTCCTCCTGCGAAGCCCCCTCTCTGGGGGCGCTGTGCAGCGAGGCCCTT-G---- 318
armadillo   ACCTTGATCTTATTTGATCCGGTCGTG-ATCCCAGCCCCGCCGGGCCGACCCGAAATGAAAAGCTGTCCTCCTGCGAAGCCCCTTCCC--GGGGCGCTGTGCAGCGAGGCCTTTTG---- 305
human       ACTTTGATCTTATTTGATCGGATCGTG-ACCCCAGCCCCGCCGGGCCGACCCGAAATGAAAAGCTCTCCTCCTGCGAAGCCCCCTCGG----GGCGCTGTGCAGCGAGGCCCCTTA---- 284
cat         ACCTTGATCTTATTTGATCGGGTTGTG-ACCCTTGCCCCGCGGGGCCGACCCGATATGAAAAGCTCCCCTCCTGCGAAGCCCCCTCCTCTGGGGCGCTGTGCAGCGAGGCCCCTTT---- 312
dog         ACCTTGATCTTATTTGATCGGGTTGTG-ACCCA-GCCCCGCCGGGCCGGCCCGAGATGAAAAGCT-CCCTCCTGCGAAGCCCCCTCCGC-GGGGCGCTGTGCGGCGAGGCCCGCG----- 331
mouse       ACCTTGATCTTATTTGATCGGGTCGTG-ACCCCCGCCCCGCCGGGCCGCCCCGAAATGAAAAGCAGCCCTCCTGCGAAGCCCCCTCGGT-GGGGCGCTGTGCAGCGAGGCCCTCTG---- 308
opossum     ACGCTGATCTTATTTGATCGGGTCGCG--CTCCTGCCCCGCCGGCCTGACCCGAGATGAGAAGCTCCCCTCCCGCGAAGCCCCGGTCG---GGGCGCTGTGCGGCGAGGCCTATGCGAAC 306
Xenopus     ACACTGATCTTATTTGATCTCCCCGCGGATGGCGGCCTCTCC---CCGAGGAGAAATGATAGGAA-ACCGCTCGCTAAGCCCTTCCCG----GGCCCTGTGCGCTGAGCTCGGCTT---- 266
Tetraodon   ACAGTGATCTTATTTGATCTGATCAGA--TTTTGGCCCTGCT---CTGATCGGAAATGATAGGAAATTCGCTTGCG-AGCCTTAATGG-CGGTGAGCAG-CCGGCGA--ACAACT----- 301
fugu        ACAGTGATCTTATTTGATCTGATCGGA--TCTGGGCCCTACT---CTGATCGGAAATGATAGGAAATTCGCTCGCG-AGCCTCCAAGG-CGTTGAGCAG-CCGGCGAGTCAAACT----- 316
            **  ***************      . .        ***    *     * *    ** **** *.*.:    * *   **   ****                *. *:*  *.   **    .

                                                                             box C     antisense box D'
rabbit      ----GGCGGCGGCAACGCCGCGGTCCCCGAGGTCCCG---GAGCTGGTCCAACGGGGCCCGGCGCTCGGAAGTGATGAATTGATCAGATAGACGAG------GCTGGGCT-TG----TC 510
armadillo   ----GGCGGTGGCAAG-CCGCGGTCCCCGAGGCCCTG---GACTCGGTCGGACGGACCCGGCGCTCAGAAGTGATGAATTGATCAGATAGACGAG------GCCGGGCT-TG----TC 405
human       ----GGCGGCGGCCACGCTGTGGTCCCCGAGGTCCCG---GAGCTGGCCCTGCGGGGCCCGGCGCTCAGAAGTGATGAATTGATCAGATAGACGAG------GCCGGGCT-TG----TC 385
cat         ----GGCGGCGGCGACGCTGCG---------GCCCCG---GAGCCGGTCTTACGGGGCCCGGCGCTCAGAAATGATGAATTGATCAGATAGACGAG------GCCGGGCT-AG----TC 404
dog         ----GGCGGCGGCAGCACCGCG---------GCCCCG---GCGCTGGTCTGACGGGGCCCGGCGCTCAGAAGTGATGAATTGATCAGATAGACGAG------GCCGGGCC-CG----TC 423
mouse       ----GGCGGTGGCGGCGTCGCGGTTCCCGGGGTTCCG---GGATCGGTCCTGCGGG-CCCGGCGCTCAGAAGTGATGAATTGATCAGATAGACGAG------GCCGGGTC-CG----TC 407
opossum     AGGCGGCGGCGGCCGCTCCCGGGTTCCCCGGAGCCAGCCCTTCGCGGGCCTGGCCGGGCCGGGCGCTCAGAAGTGATGAGTTGATCAGATAGACGAG------GCGGGGCT-TG----TC 415
Xenopus     ------CGGAGACCGCAAATC----------------------------------------------ACTGAAAGGATGAATTGATCAGATATACTAG------GCCGGGCTGCGGGGCTC 329
Tetraodon   ------CTGGGGCCTCCGCAAT----------TCATG-----TTGTGG------------------TCAGAGGTGATGAATTGATCAGATATACTAGTTCTTTGTGTGTTTGGGTTCATC 382
fugu        ------CCAGGGCCTCCACGCT----------TTAAG-----TTGTGG------------------TCAGAGGTGATGAATGGATCAGATATACTAGTGTTCTGTGGGTTCGGG-TCATC 396
                  *  .  *.*                                                   * **.. *****.* ********* ** **       *    *      *    **

                            box C'          box D
rabbit      CCG-----------GGCCGCTGATTATCGAGGCGATTCTGATCTGA 454
armadillo   CTG-----------GGCCACTGATTATCGAGGCGATTCTGATCTGA 440
human       CCC-----------GGCCACTGATTATCGAGGCGATTCTGATCTGG 420
cat         CCG-----------GGCCACTGATTATCGAGGCGATTCTGATCTGA 439
dog         CCG-----------CGCCGCTGATTATCGAGGCGATTCTGATCTGA 458
mouse       CC------------AGCCGCTGATTACCGAG-CGATTCTGATCTGA 441
opossum     CC------------TGCCCTTGAGTATCGAGGCGATTCTGATCTGA 449
Xenopus     CCCCATAATCCC--GGCCTGTGAGTATAGAGGCGATTCTGATCTGA 373
Tetraodon   TCAAATCTCACTGTGGACAATGAGTATAGAGGCGATTCTGATCTAA 428
fugu        TCGAACGTTACGGAGGACAATGAGTATAGAGGCGATTCTGATCTAA 442
                           *.*  *** ** .*** ************..
```

Figure S4. Alignment of vertebrate mgU2-25/61 RNAs.

```
human           ---AGAGGCTTGGGCCGCCG----AGCTGG-------ACCCGGACCGG-----------TTTTGGG-TACTGTACTGGG--GGCAGGGCA----GAGAGG--------TGGGCG------ 74
cat             ---CTGGGTTTGAGAGAGAC----CCTAGG-------ACTGGAAGCTA-----------GTTGGCG-GGCCCGAGTGGGGTGGTGGGGTG----GGGAGG--------TTGGCG------ 76
armadillo       ---AGATCAGTGGGCAGAGG----CCTAGA-------TCTCTTCGCCG-----------GACGAAGGAGGCGTGGTGGGCGGGAGGCATGGT--GGGTGG--------GAGGCGAAGAGG 85
rabbit          ---ATAGTATTTCTCAATGGTGACACTTGG--GAATTTGCAGAAGCTG-----------CTTTGGAGTTCCAGGTGGCG--GGCGTGGGC-GCGGGGCAG-------GCCGGCGGG---- 90
dog             ---AGAGGGCGGCGCCGCGGGATTGGCTGGGCGCCCGTGCGGGGGCGGGGCGGGGCGGGCGCGGCTGCGCGGAGGGGCGGAGGCCTAGGCTGCGCGGCGGGAGAGGGGCCGGGAGGA--- 114
mouse           ---GGTGGGATTGGCTGCCCTTGCGCCTGCGCAGCCTTCTGGCGGCGG----------CCTGTGCTAGGTCGG--AGCGG-GGCGGTGGC----GGGTAG--------CTGGGGCG---- 88
opossum         ---CGGGGCCTGGGACAGTG--ATAACT---------TCCGGGAGCGC------------GTTGCTTTGTCCTCAGACT--CGCCTCGGC-----GGTCT--------TTGGGGG----- 74
lizard          --ATGGTCCCCAAACGAAAGCATTCCTTGG-------GTGGTGGGCCG-------------TAGTGTTTGGGAAGGACATTGGCAGGGGT---TGGGCTG--------CATATT------ 81
turtle          ---TAAAGCTTTATATCTGTTAAAACAC----------AAATTGTCAA-------------CATCA--CATATCAGAATATGCAAAGTAA-----ATAT-------------------- 66
Coelacanth      GTCTAAGGGTTTTTTATTATTTATTTTGG-------TAACTTTTCAG-------------TTTTAGAAGTGTTTTACAATACAATACAA-----ATTTA------------------- 75
                                                             *
                                                                                                box C          antisense  box D'
human           ---GCAGTTGGGGTGCGGTG-----ATTGTAGTAGGCTAGGG-CGCTTTCGGGTC--CCCATTGCAGC--------CCCCGGATGAGCCCGCAGTATTTTCCTTATATGATCAGGTCCCA 175
cat             ---TCCGAGGGAGTGTAGTG-----AGTGTCTTAGGGCAGGG-ATGCCCCGAGTC--CCCGAAATAGC--------CCC-CGATGAGCTCGCAGTATTTTCCTTATATGATCAGGCCCCG 176
armadillo       TTGGCTTCGGGGGTGTAGCG-----AACGTAGTAGGTTAGGGGCGCCCGGGGATC--CCTGATCCAGC--------CCCTGGATGAGCCCTCAATATTTTCCTTATATGATCGGGCCTTT 190
rabbit          ----CGGGTGGGGTGCGGCG----AGGGTTG-TAGGGTAGGG-TGCGCCCGGATC--CCAAGTGCAGC--------CCCCGCATGAGCCCGCAGTGTTTTCCTTATATGATCAGGCCCCG 190
dog             ---CCGGGTGGGGGGCGGTGGTC-GGGGTTGGAAGCGCGGGA-AGCCCCCGAGTC--CCCAGTGTAGC--------CCCCTCGTGAGCTCGCGGTATTTTCCTTATATGATCGAGCTCCG 219
mouse           ---TCTAGAGAAGGGAAGGG----GAACGTGGTAGAGCAGGG-AGCGCTTGGGTC--CCCGAGGCAG---------CCCCGAGTGAGCCCGATGTATTTTCCTTATATGATCAGGCCTCT 189
opossum         -----AAGGAGGATTCTTTG----CAGTTTAGGAAGGGGGAGAAGCGCCCGAGTCGCCCCAACGCAGC--------CCCCAAGTGA-CCCACTATATTTTCCTTATATGATCGGGCTTCT 176
lizard          ----CATGGAGCTCGCTGTAACCACAATGTCAATGGAAAAGATTGACTTGGTGGCTCCTCAGCACTGGGAACTGTAGCCTATTTGTGAAGGCCGGAGGCTGTTTGTGTGAACAGACACCC 197
turtle          ---CCTTAAATCAAATTGAG----TTCTCAAGCAGCATTTTTTACTTTGTCTATCTGTAAATTTAGAT--------GATCATAGATGGAAATATTTTAAT--TGTTTTGTGCT-CATACA 168
Coelacanth      ---CCTGAAGTAAGTTTGTG----TTGTGTTGTAAAACATTG-CTTAAGGCTGGGAACGATATTCAGT--------GAAAAAAAAAATACTTTGTAATATTCTGTAATAGGCT-AAAATT 178
                         .         .         :   :.                 .             .          .      .:             *  *  : *.  *
                                                          box C'                        box D
human           TTGCGGGCGGCGCCGCTTGCC---------CGGAGCCTGAGAGGATTATG----------AAAACGTGGCGAGCGAAATGGGGCCAGGGGACCTGGAG-----CAGGGGCGTGAGGAGAG 271
cat             TTGGTGGCGACGCCGCTAGCC---------CGGAGCCTGAAAAGATTATG----------AAAATGTGTCCGGCGAAGTGGGG-CTGGGGACCGGGAG-----CCGGGAGATTAGG---- 267
armadillo       TTGTAGGCGGCGCCGCCTGCC---------CGGAGCCTGAGAGGATTATG----------AAAAT-TGCCGGGTGATGTGGGGCCAGGGGACCGGGCG-----CGGGGAGGTAGAGGGCA 285
rabbit          TTACGGGCGGGGCCGCTTGTCT--------CAGGGCCTGAGAGGAATATG----------AAAACGAGGCAGGCGAAGTGGGG-CTCGGGGCCCG----------GGCAGTTAGGGGCGA 281
dog             CT-----CGGCGCCGCTGGGC---------CGGAGCCCGAGAGGATTATG----------AAAACGTGTGGGGTGAGGTGGGG-CTGGGGACCGG----------GGCG-----GGGAGA 299
mouse           CG-TAACCGGCGCTGCTGGTT---------TGGAGCCTGAGAGGATTATG----------AAAACGTGGCAGGTGATG-GGGT-CAGGGGACCTGGAG-----CGGGGAAGCAGAGGGGA 282
opossum         GGGCAGGGGACGGTTTGGTACT--------GGGAGCCCGAAGTGATTATAGA--------AAAATGATGCAGGGGATGGGAGGCTTGGGGCCCGGACTGGCGCTGGGCAGCAAGGCAGGG 280
lizard          GTGCCACATACACACATATACAGGTTTTTACTAGATCTGTATGTCTAGTCTCGTCTAGACTAGACTAGACTAGATATTTAGATATAGATTGCATTCAGCATGGAGGTAACTTAAGGGTGT 317
turtle          GTGAAATTGACATTCACCAAC----------ATTTACTGATAAAAATCT-----------AATCCTAGTT--CTAATGTGACTGGGAGAATGACTGGT------GGGAACATCTTGCAT- 258
Coelacanth      AT-TTATTTATTTGGGTTTTT----------AGGGACTGTGTAAAGATAA----------CTTAATCGGTGGTTAATTTAGATAGAAGAGACATAGAT------AAGTTAAAGTGACATT 271
                         .                          * * .   . :              : .           *   ..      .    .            .
                                                                                                               box C      anti-
human           TAGGCAG------------CGGGTGAGGCTGG-------ACGGGAGGGAGGTCTAG-----------GGAGGCCTCTGCCGCGGGCACTGTGAGTCCTGGCCGATGATGACGAGACCACT 361
cat             --GGTAG------------CGGGT-AGGCTGG-------AGATGAGGGAGCTTTAG-----------GAAGGCCTCTGCCGCTGTAACTGTGAGCCCTGGTCGATGATGACGTGACCACT 354
armadillo       AGGGTAGT-----------CGGGT-AGGCCGG-------AGAGGCGGAAGGTTTAG-----------GGAA-CCTCTGCTGCTG------TGAGCCCTGGTCGGTGATGACGTGACCACT 368
rabbit          GAGGGGCG----------GTGGGG-AGGCCT--------------------------------------CTGCCACTGTGACCG------TGAGCCCTGGCCGATGACGACCAGACCACT 346
dog             CCGGGCGG----------TCGGGG-AGGCCC--------------------------------------CTGCC-CTGCAGCGG------CGAGCCCGGGTCGATGATGACGTGACCACT 363
mouse           GAGGGACTGA--------GTGGGG-AGGCCAGGCTTCGGGGAGGGGATAGGTTTAGGGG-------TACCCACGCCTCCAGCTG------CAAGCCCTG-CCAATGATGACATGACCACT 379
opossum         CAGGGAAGGGGCAGCTTCCTGGGCTGTGCCCCTGGGGAGGGTGGGCGCTGCTGGGGGTGGGGGAGGAAGGGGAGCCATAGGCGGCTCTTTGGAACCCTGGTCGATGATGACGTGGCCACT 400
lizard          TTGGTTGTGGCTACAATTTTAGGATGTAATTTG--TCATACGTTCAAATACTCCAGTTAG-------AGAAGCATTTCCCCTTG---TTGCAAGGACTGGTCTGTGATGAATAGCCCACT 425
turtle          GAAGATGG-----------CAGGAAAG-------------------------------------------AAAAAGTGTGGAGCTC----ACTAAGCCCTGGTCTGTGATGATTAGCCCACT 322
Coelacanth      AAAAATGG-----------TAGGAATGGATTTT-----------------TTTTT-------------AAATTGTGTAGTAATT----TTAAACCAATGGTCTATGATGACTAGCTCACT 346
                  ..                .**                                                     :              .*  ..  *  * .*** **   :* ****
                 sense box D'                  box C'                     box D
human           GCGCAATCTGAGTTCTG---GGAACC--AGGTGATGGAGTATGTTCTGAGAACA--GACTGAGGCCG 421
cat             GCGCAATCTGAGTTCTA---GGAACC--AGGTGATGGAGTGTGTGCTGAGAGCT--GACTGAGACCG 414
armadillo       GCTCAATCTGAGTTCTT---AGAACC--AGGTGATGGAGTGTG-GCTGAGAATG--GACTGAGACCG 427
rabbit          GCACAACCTGAGTCCTG---CACACC--AGGCGTTGGAGTGTGTTCTGAGAACA--GACCAAGGCCG 406
dog             GCGCAATCTGAGTTCTAG--GAACCC--AGGTGATGGAGTGGATGCTGAGAGCG--GACTGAGACCT 424
mouse           GCGCAATCTGAGTTCTG---GGAACC--AGGTGATGGAGTG--TGCTGAGAAC---ACTGAGGCCA 435
opossum         GCGCAATCTGAGTTCGT---AGAACC--AGGTGATGGAATGTGTGCTGAGAGCG--GACTGAGACCA 460
lizard          GCGCAATCTGAGTTCTGT--AGAACC--CAATGATGGATTTTGTGCTTTCGAAGGCATCTGAGACCA 488
turtle          GCGCAATCTGAGTTCTGC--AGAAC--TACATGATGAGATTTGTGCTGAGAAAGGCAGCTGAGACCA 385
Coelacanth      GCGCAATCTGAGTTCTGCGGAGAACGTATCATGAGAGAAGCAGTGCTGACGAAG--AACTGAGACCA 411
                ** *** ****** *      ...*     . *: ...      ** : ..     *.**.**
```

Figure S5. Alignment of vertebrate mgU12-22/U4-8 RNAs.
